# Importance of Meta-analyses for Evaluating Carcinogens

**DOI:** 10.1289/ehp.1104755

**Published:** 2012-04-01

**Authors:** Manolis Kogevinas, Neil Pearce

**Affiliations:** Centre for Research in Environmental Epidemiology (CREAL), Barcelona, Spain, E-mail: kogevinas@creal.cat; Faculty of Epidemiology and Public Health, London School of Hygiene and Tropical Medicine, London, United Kingdom

Camargo et al. (2011) published the most complete meta-analysis on asbestos and ovarian cancer, concluding that “Our study supports the IARC [International Agency for Research on Cancer] conclusion that exposure to asbestos is associated with increased risk of ovarian cancer.” Imagine if it had not!

The IARC Monograph Working Group met in 2009 (Straif et al. 2009), but the working group, which included four authors of the Carmargo et al. (2011) paper, apparently did not make use of a formal meta-analysis of all available studies to evaluate the evidence. Although the *IARC Monographs* staff have occasionally conducted meta-analyses prior to a monograph (e.g., Guha et al. 2009), formal meta-analyses are not prepared routinely for the evaluations. However, such meta-analyses can be considered when they are publicly available and, as stated in the “Preamble to the *IARC Monographs*,” “ad-hoc calculations that combine data from different studies may be conducted by the Working Group during the course of a *Monograph* meeting” (IARC 2012).

Although meta-analyses have been widely used in social sciences and medical research for decades, it is only recently that they have become more widely used in epidemiology (Greenland and O’Rourke 2008), and they were not widely used at the time that the *IARC Monograph* procedures were first developed. As Greenland and O’Rourke (2008) comment,

Epidemiologic studies of specific topics have tended to be fewer [than is the case in social science or medicine], and the epidemiologic community appears to be more hospitable to tentative, limited inferences based on narrative reviews. Nonetheless, to neglect quantitative aspects of review would be akin to presenting a study and supplying only a narrative discussion of the raw data, with no attempt to group and compare subject outcomes.

Having participated in several Monograph Working Groups, we know that informal meta-analyses are performed by members of the working groups (at least by ourselves) because this is one of the standard ways to quantitatively summarize the evidence. Meta-analyses can be challenging and controversial depending on assumptions, particularly for studies on occupational and environmental exposures that frequently are not easy to quantify. Certainly meta-analyses cannot be done for all agents evaluated by IARC, and results of meta-analyses have to be critically interpreted in a manner similar to that of all other data.

Even without using formal procedures for a quantitative summary of the epidemiological evidence, IARC Working Groups reach conclusions that are seldom seriously challenged, particularly for Group 1 carcinogens. This is probably because IARC Working Groups tend to express the consensus in the scientific world. When they occasionally have made controversial decisions, for example the 1997 dioxin evaluation reclassifying TCDD from a Group 2B (possible) carcinogen to Group 1, they seem to have been proven right by later research. IARC has revised procedures for the *Monographs* evaluations, incorporating the systematic inclusion of biological knowledge into the criteria for the classifications, and has also updated the whole process for other items, such as conflict of interest and the prodecure for the selection of participants. These changes have strengthened the validity and acceptance of these evaluations. Given the international importance of the *IARC Monographs,* it seems advisable to use all the tools available for summarizing the scientific evidence, one of which is meta-analysis. The meta-analysis by Camargo et al. (2011) clearly demonstrates this regarding both the overall meta-estimate that is not negligible (standardized mortality ratio of around 1.8) and the discussion of the heterogeneity of the findings between studies that can be formally examined in a meta-analysis. If we had been members of that Working Group, we would certainly have preferred to have this meta-analysis on hand before doing the evaluation.
